# A case report of *CIC–DUX4* fusion-positive sarcoma in the pelvic cavity with targeted next-generation sequencing results

**DOI:** 10.3389/fonc.2022.1018992

**Published:** 2022-12-15

**Authors:** Qian Wu, Ying He

**Affiliations:** ^1^ Department of Pathology, West China Second Hospital of Sichuan University, Chengdu, Sichuan, China; ^2^ Key Laboratory of Birth Defects and Related Diseases of Women and Children (Sichuan University), Ministry of Education, Chengdu, Sichuan, China; ^3^ NHC Key Laboratory of Chronobiology (Sichuan University), Chengdu, Sichuan, China

**Keywords:** *CIC–DUX4*, undifferentiated small round cell sarcoma, target next generation sequencing, low tumor mutational burden, *POLE*

## Abstract

*CIC–DUX4* fusion-positive sarcoma is a subtype of undifferentiated small round cell sarcoma that is rarely reported. As far as we know, less than 200 cases have been reported worldwide to date. The clinicopathologic characteristics of this kind of tumor are non-specific, which makes it difficult to be diagnosed. Therefore, more cases are required to enrich the diagnosis and treatment experience. Here, we present a 17-year-old Asian girl diagnosed with *CIC–DUX4* fusion-positive sarcoma after targeted next-generation sequencing. Her clinical manifestation was abdominal pain. Furthermore, a mass in the pelvic cavity and massive ascites were found after an imaging examination. After resection, the mass was sent to the pathology department for a definite diagnosis, and the micromorphology showed an undifferentiated sarcoma with massive necrosis. The tumor cells were round to spindle with clear to eosinophilic cytoplasm and vesicular nuclei. Rhabdoid cells and myxoid mesenchyme were focally shown. Immunohistochemical staining showed diffusely positive for vimentin, cyclin D1, Fli-1, and WT-1 and very focally positive for CD99. Moreover, the targeted next-generation sequencing also revealed other genetic changes in this tumor including LongInDel of *POLE*, copy number variation of *CD79*, low tumor mutational burden, and microsatellite stability. With a follow-up time of 6 months, the patient survived the disease and received chemotherapy routinely. This report presented a rare primary site CIC–DUX4 fusion-positive sarcoma (CDS) and revealed novel genetic changes that enrich the manifestation, histology, and cytogenetic scales of this rare sarcoma. In addition, we have summarized the clinicopathologic characteristics of this tumor by reviewing the literature to have a better understanding of *CIC–DUX4* fusion-positive sarcomas, which may be helpful for diagnosis and treatment.

## Introduction

Undifferentiated small round cell sarcomas (USRCSs) are a group of tumors with similar morphology, which makes it difficult to identify and diagnose. However, with the rapidly developing molecular pathology, it is possible to clarify them by genetic changes. *CIC*-rearranged sarcoma is one of the new tumor types of the USRCS, which used to be considered Ewing-like sarcoma.

Somers et al. (1) presented a case of an unusual and more aggressive cutaneous and subcutaneous primitive neuroectodermal tumor/Ewing sarcoma (PNET/ES), which showed a complex karyotype with t(4;19)(q33~q35;q13.1). Two years later, Kawamura-Saito et al. (2) found a recurrent chromosomal translocation t(4;19)(q35;q13) in two cases and recognized the fusion of *CIC–DUX4*. Afterward, Italiano et al. implicated that *CIC* was fused to copies of *DUX4* gene not only on 4q35 but also on 10q26.3 (3). Moreover, more fusion partners of *CIC* were found, such as *FOXO4* (4, 5) and *NUTM1* (6, 7). More cases were included to summarize the characteristics of this kind of tumor and showed a distinct immunoprofile, karyotype, and worse outcome as compared to ES (8, 9), suggesting that it was a new pathologic entity.

To date, more than 200 cases of *CIC*-rearranged sarcoma have been reported, among which the *CIC–DUX4* was the most common fusion (1–3, 7–32). Here, we report a case of *CIC–DUX4* fusion-positive sarcoma (CDS) arising in the pelvic cavity of a 17-year-old girl with targeted next-generation sequencing results, which enriches the manifestation, histology, and cytogenetic scales of this rare sarcoma.

## Case report

**Figure 1:**
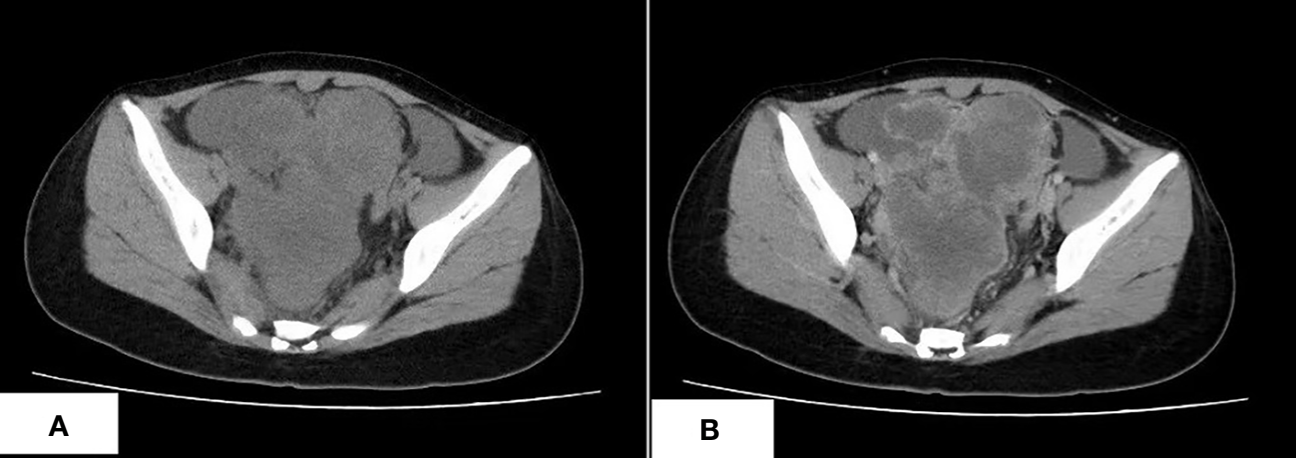
The CT scan of CDS. **(A)** An irregular lobulated mass in pelvic cavity, which has unclear margin with peripheral structure, is shown. **(B)** The tumor shows heterogeneous enhancement in the contrast-enhanced CT scan.

A 17-year-old Asian girl came to the hospital with complaints of abdominal pain. A computed tomography (CT) scan showed a cystic and solid mass measured 12.1 cm × 8.2 cm × 13.6 cm in the pelvic cavity with heterogeneous enhancement in an enhanced scan (**Figure 1**). Ultrasound detected fluid dark areas in multiple abdominal spaces. The serum level of carbohydrate antigen 125 was 447.6 U/ml. After adequate evaluation and preparation, the patient received surgery. During the surgery, ascites with a volume of 3,000 ml were drained from the abdominal cavity. Both the mass and ascites were sent for pathologic examination.

**Figure 2:**
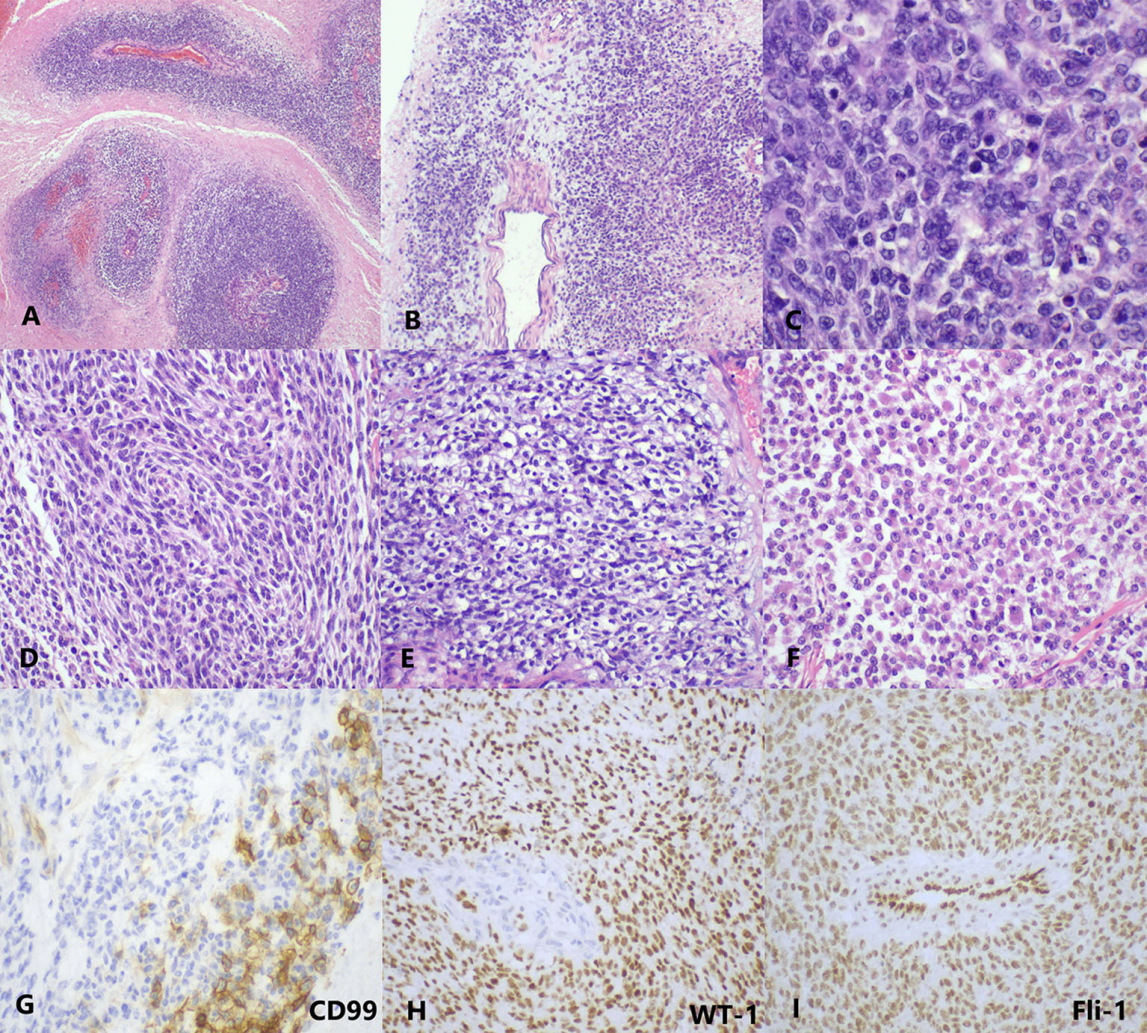
The histopathology and immunohistology of CDS. **(A)** At low magnification, the tumor cells are separated by massive necrosis, arranged in nest-like and perivascular (H&E, ×40). **(B)** Focal myxoid stroma (H&E, ×40). **(C)** At high magnification, the tumor cells show round-to-oval shape with lightly eosinophilic cytoplasm and vesicular nuclei; several mitotic figures are visible (H&E, ×400). Moreover, **(D)** spindle cell-like (H&E, ×200), **(E)** clear cell-like (H&E, ×200), and **(F)** rhabdoid plasmacytoid tumor cells (H&E, ×200) are also exhibited in some areas. **(G)** CD99 is focally membranous positive (IHC, ×400). **(H)** WT-1 (IHC, ×200) and **(I)** Fli-1 (IHC, ×200) are diffusely nuclear positive. IHC, immunohistochemistry.

Grossly, the tumor showed a fused-nodular mass with an appearance of color and a touch of tenderness when being cut. The histological examination demonstrated the tumor consisted of round to spindle malignant cells with clear to eosinophilic cytoplasm and vesicular nuclei. Rhabdoid cells and myxoid mesenchyme were focally shown. Moreover, necrosis and pathologic mitoses were apparent (**Figures 2A–F**). Tumor cells were also detected in the peritoneal lavage. Immunohistochemical (IHC) staining showed diffusely positive for vimentin, Fli-1, cyclin D1, and WT-1 and very focally positive for CD99 (**Figures 2G–I**). Pan-CK, ER, PR, CD117, DOG-1, NSE, Syn, CD34, S100, desmin, caldesmon, CD10, and Sall-4 were all negative. The index of Ki-67 was >90%.

**Figure 3:**
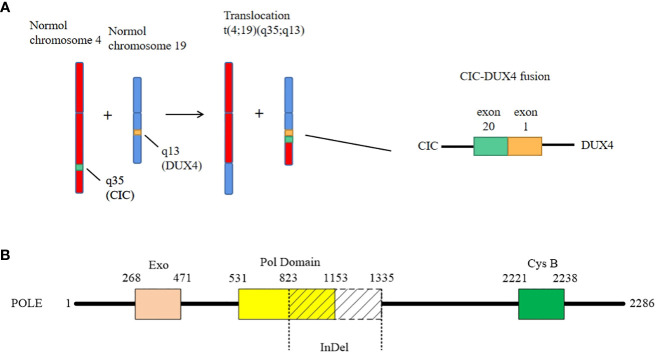
The genetic changes of the case. **(A)** The case shows a classical fusion of *CIC–DUX4*, which is generated by translocation of chromosome t(4;19)(q35;q13). The breakpoints are located in exon 20 of *CIC* and exon 1 of *DUX4*, respectively. **(B)** The LongInDel of *POLE* (exon22_exon31del) causes deletion of amino acid residues 823–1,335 of POLE protein, which may lead to frameshift and loss of polymerase domain 25 and CysB motif (amino acid residues 2,221–2,238).

For diagnosis and further treatment strategy, the paraffin section of the tumor was sent for targeted next-generation sequencing (NGS) on the Illumina platform. The exons of 672 and 633 genes were detected by DNA sequencing and RNA sequencing, respectively. The results were as follows: 1) fusion between *CIC* exon 20 and *DUX4* exon 1 (**Figure 3A**), 2) LongInDel of *POLE* (exon22_exon31del) (**Figure 3B**), 3) copy number variation (CNV) of *CD79A* (5.8 copies), 4) low tumor mutational burden (TMB) at 0.5 Muts/Mb, and 5) microsatellite stability (MSS). Finally, this tumor was diagnosed as CDS.

After the surgery, the patient went to another medical institution for further treatment. At a follow-up of 6 months, the patient was still alive.

## Discussion

In the latest (2020) World Health Organization (WHO) classification of tumors of soft tissue and bone, *CIC*-rearranged sarcomas were categorized as a new entity of USRCS (33). So far, less than 200 cases have been reported, including 157 cases of CDS, among which 88 cases are available for clinicopathologic information. The age ranged from 8 to 69 years (average at 30.7 and median at 29.5). Gender, 41 male (46.6%) and 47 female (53.4%), did not yield much difference. The most common primary sites are the soft tissue of the limbs (43/88, 48.9%), followed by the soft tissue of the trunk (35/88, 39.8%) and parenchymal organs (7/88, 7.9%). Only three cases originated from bones (3/88, 3.4%) (18, 26), which was distinguished from ES. Only two cases arose from the pelvic cavity: one is a 45-year-old man, and the other is the current case report. The maximum diameter of tumors was mentioned in 35 cases, which ranged from 1.5 to 20 cm (mean at 8.2 cm and median at 8 cm). The manifestations are usually not special, mostly occasionally discovery of mass. However, some unique manifestations are also reported, such as mimicking a phlegmon (27), cardiac tamponade (31), vaginal bleeding (28), and massive ascites in this report, which are caused by the tumor invasion of particular organs. The most common metastasis organ of CDS is the lung, followed by the brain and liver.

For morphology, the typical CDS usually consists of undifferentiated small round-to-oval tumor cells with clear to eosinophilic cytoplasm and vesicular nucleus, arranged in sheets and nests. Pathologic mitoses and geographic necrosis are easy to see. Focal myxoid matrix (1, 3, 7–9, 11, 13, 14, 18, 25, 26, 30) and spindle tumor cells (3, 8–11, 14, 18, 19, 21, 27) are also reported in some studies. Other less common morphologies include rhabdoid appearance or plasmacytoid appearance (3, 9, 12, 18, 19, 25, 32), epithelioid (18, 32), formation of microcystic spaces or pseudoacinar arrangements (8, 9, 15, 21, 23, 32), or single cell cords (13). There is one case in that focal hyaline cartilage formations appeared after chemotherapy (19).

During immunohistochemistry, almost all CDS showed vimentin positivity, and most showed weak and focal membranous CD99 positivity. Specht et al. (8) found that WT-1 was strongly positive in all CDS cases but negative in all ES cases. Therefore, they proposed that consistent WT-1 expression might provide a useful clue in the diagnosis of CDS. However, it may be a pitfall in diagnosis when CDS is primarily in the kidney. There is a report that a renal CDS was originally diagnosed as a stromal Wilms tumor (WT) because of the expression of WT-1 and rare recognition of CDS (21). In addition, CDS also frequently expresses Fli-1 and ERG, the ETS transcription factor family members (14). Cytokeratin is usually very focal positive. Bcl-2 positivity (3, 20, 21), ETV4 positivity (24), and C-MYC protein expression (15) were also reported. Moreover, Yoshimoto and his colleagues found that cyclin D2 and MUC5AC were novel biomarkers and were useful for distinguishing CDS from ES (34).

For diagnosis, although clinical, histological, and immunochemical characteristics of CDS are well recognized, none of them are entirely sensitive or specific. Therefore, a molecular testing result of *CIC–DUX4* fusion is necessary to reach the final diagnosis. Fluorescence *in situ* hybridization (FISH) and targeted RNA sequencing are the most popular methods to detect *CIC* rearrangement. However, Yoshida et al. described four cases that showed negative results by CIC break-apart FISH assays but identified *CIC–DUX4* and its fusion breakpoint through high-throughput RNA sequencing (24), suggesting there may be a false-negative rate for FISH to detect CDS. Moreover, ETV transcriptional upregulation (22), DNA methylation profiling, and next-generation sequencing (29) were suggested to be more sensitive than FISH and RNA sequencing. Therefore, when histology and immunoprofiles highly indicate the diagnosis of CDS but the result of FISH or RNA sequencing is negative, conclusions must not be drawn yet; it is necessary to consider another detection technique.

For pathogenesis, Kawamura-Saito and his colleagues found that *CIC–DUX4* protein transforms NIH 3T3 cells and is a strong transcriptional activator that upregulates *PEA3* family genes (2). Other researchers revealed that *CIC–DUX4* sarcomas demonstrate frequent *MYC* amplification and ETS-family transcription factor expression (14). These studies uncovered the downstream targets of *CIC–DUX4* protein. Subsequently, the upstream factors were found by Lin et al. (35), who suggested that negative *MAPK-ERK* regulation sustained *CIC–DUX*4 oncoprotein expression. Moreover, targeted NGS was also performed to characterize potential somatic driver alterations of CDS. Vega et al. analyzed 11 CDS cases by targeted NGS. Although no recurrent somatic mutations were identified, they detected low mutational burden and recurrent chromosome 1p loss (36). Other researchers also found a low mutational burden by targeted NGS, as well as stable microsatellite status (28), which is consistent with this report. However, we found mutations of *POLE* and *CD79A*, which have not been reported before. Moreover, Ricker and her colleagues noticed the overexpression of the translation factor *eEF1A1* when whole-genome sequencing and RNA sequencing were performed in a case of CDS (30) (**Table 1**). Furthermore, cell lines were established for drug sensitivity tests, and bortezomib, crizotinib (37), and inhibition of the CCNE–CDK2 complex (38) seemed to be able to suppressed the growth of CDS cells. However, more studies are needed to better understand this rare sarcoma.

**Table 1:** The summary of studies about next-generation sequencing (NGS) of *CIC–DUX4* sarcomas.

Authors	Case(s)	Methods	Discoveries
Vega et al.	11	Targeted NGS (409 genes)	1) Low mutational burden 2) Recurrent chromosome 1p loss 3) *ARID1A* R963X non-sense mutation in a recurrence sample
Sedighim et al.	1	Targeted NGS	1) Low mutational burden 2) Stable microsatellite status
Ricker et al.	1	Whole-genome sequencing and RNA sequencing	Overexpression of the translation factor *eEF1A1*
This report	1	Targeted NGS (672 genes) and RNA sequencing (633 genes)	1) *POLE* exon 22–exon 31 LongIndel 2) *CD79A* copy number variation (CNV) 3) Low mutational burden 4) Stable microsatellite status

NGS, next-generation sequencing.

What is worthy to be mentioned is that we found a novel mutation of *POLE.* It is well known that exonuclease domain mutations of *POLE* were mostly reported in some epithelial tumors such as colorectal cancer and endometrial carcinoma, which lead to a deficiency of proofreading activity and usually high TMB (39). However, in this case, the mutation of *POLE* is LongInDel from exon 22 to exon 31 (823–1,335 amino acid residues), which causes the loss of part of the polymerase domain and CysB motif (2,221–2,238 amino acid residues) (**Figure 3B**). The site of mutation is beyond the exonuclease domain, which means it is a non-proofreading mutation. A study suggested that tumors with *POLE* proofreading mutation showed a significantly higher TMB than tumors with non-proofreading mutations (40), which may explain why this sarcoma has a *POLE* mutation but low TMB. Moreover, we revealed the CNV of *CD79A*, which may lead to the increase of its mRNA and protein expression levels and influence the B lymphocyte antigen. However, the significance of these mutations needs more exploration in the future.

## Conclusions

We report a case of CDS arising from the pelvic cavity, which is a rare primary site for the tumor. Moreover, the results of the targeted NGS, which is LongInDel of *POLE* and CNV of *CD79A*, were the first to be reported. Therefore, our case enriches the clinical and genetic scales of CDS.

## Data availability statement

The original contributions presented in the study are included in the article/supplementary materials. Further inquiries can be directed to the corresponding author.

## Ethics statement

Ethical review and approval were not required for the study on human participants in accordance with the local legislation and institutional requirements. Written informed consent was obtained from the minor(s)’ legal guardian/next of kin for the publication of any potentially identifiable images or data included in this article.

## Author contributions

QW wrote the manuscript. YH diagnosed the case and revised the manuscript. Both authors contributed to the article and approved the submitted version.
